# Overexpression of rice F-box protein *OsFBX322* confers increased sensitivity to gamma irradiation in *Arabidopsis*

**DOI:** 10.1590/1678-4685-GMB-2018-0273

**Published:** 2020-03-02

**Authors:** Jung Eun Hwang, Sun-Goo Hwang, In Jung Jung, Sung Min Han, Joon-Woo Ahn, Jin-Baek Kim

**Affiliations:** ^1^ National Institute of Ecology, Research Center for Endangered Species, Division of Restoration Research, Yeongyang, Republic of Korea.; ^2^ Kangwon Natl University, Department of Applied Plant Sciences, Plant Genomics Lab, Chuncheon, Republic of Korea.; ^3^ Korea Atomic Energy Research Institute, Advanced Radiation Technology Institute, Jeongeup, Republic of Korea.

**Keywords:** F-box protein, gamma-irradiation, ionizing radiation, rice, transgenic plant

## Abstract

Ionizing radiation has a substantial effect on physiological and biochemical processes in plants via induction of transcriptional changes and diverse genetic alterations. Previous microarray analysis showed that rice *OsFBX322*, which encodes a rice F-box protein, was downregulated in response to three types of ionizing radiation: gamma irradiation, ion beams, and cosmic rays. In order to characterize the radiation-responsive genes in rice, *OsFBX322* was selected for further analysis. *OsFBX322* expression patterns in response to radiation were confirmed using quantitative RT-PCR. Transient expression of a GFP-OsFBX322 fusion protein in tobacco leaf epidermis indicated that OsFBX322 is localized to the nucleus. To determine the effect of *OsFBX322* expression on radiation response, *OsFBX322* was overexpressed in *Arabidopsis*. Transgenic overexpression lines were more sensitive to gamma irradiation than control plants. These results suggest that *OsFBX322* plays a negative role in the defense response to radiation in plants. In addition, we obtained four co-expression genes of *OsFBX322* by specific co-expression networks using the ARANCE. Quantitative RT-PCR showed that the four genes were also downregulated after exposure to the three types of radiation. These results imply that the co-expressed genes may serve as key regulators in the radiation response pathway in plants.

## Introduction

Abiotic factors such as drought, salinity, temperature, and ultraviolet (UV) light can influence plant growth and development and limit cultivated plant productivity worldwide. Plants respond and adapt to these stresses, and achieve stress tolerance through various biochemical and physiological processes, including those involving changes in gene expression (Kawasaki *et al.*, 2001; Seki *et al.*, 2001; Hazen *et al.*, 2003).

Plants are often exposed to a range of DNA-damaging agents from the extracellular environment, such as UV light, solar radiation, and ionizing radiation. Ionizing radiation affects plant growth and development in a variety of ways. These include stimulatory effects at very low doses, increasingly harmful effects on vegetative growth as doses increase to medium levels, and pronounced decreases in reproductive fitness and yield at high radiation levels (Prithivirajsingh *et al.*, 2004). In radioactively contaminated environments, plants, due to their sessile nature, are directly affected by harmful radiation and must adapt to life in ionizing radiation. Therefore, plants deserve special attention relative to their susceptibility to ionizing radiation. Ionizing radiations are classified according to their linear energy transfers (LETs). Alpha particles, neutrons, and heavy ion beams exhibit high LETs, whereas gamma rays, X-rays, and electron beams exhibit low LETs (Yang and Tobias, 1979). Cosmic rays are generated by the solar system and are composed of high-energy particle radiation (Sharma, 2008). These types of ionizing radiation were reported to affect stress responses in a similar fashion to other environmental stresses (Hosoki *et al.*, 2012); however, the mechanisms underlying plant responses to ionizing radiation remain largely unknown. Our recent microarray study identified many novel ionizing radiation response genes, including 53 genes that were expressed specifically in rice (Hwang *et al.*, 2014). One of these genes, *OsFBX322*, which is predicted to encode an F-box domain containing protein, is investigated further in this study.

F-box proteins, which contain a conserved F-box domain (40–50 amino acids), comprise one of the largest protein families, with approximately 700 members in *Arabidopsis* and rice (Gagne *et al.*, 2002; Jain *et al.*, 2007). Numerous F-box proteins were previously identified as components of SCF complexes (SKP1-Cullin-F-box), but non-SCF-complex F-box proteins were also noted (Clifford *et al.*, 2000; Galan *et al.*, 2001; Kim *et al.*, 2002). Only a small proportion of the known F-box proteins have been studied in plants to date. These plant F-box proteins were shown to play important roles in regulating various developmental processes and mediating stress responses via integration of almost all the phytohormone signaling pathways (Lechner *et al.*, 2006; Dreher and Callis, 2007; Zhang *et al.*, 2008; Yan *et al.*, 2011). The functions of only three F-box proteins have been described in rice. GID2 (GA-insensitive dwarf 2), the first F-box protein identified in rice, is involved in positive regulation of gibberellic acid signaling (Sasaki *et al.*, 2003), D3 (dwarf 3) F-box proteins are involved in tiller bud activity (Ishikawa *et al.*, 2005), and MAIF1 (miRNAs regulated and abiotic stress induced F-box gene) is proposed to play a negative role in the response to abiotic stresses by regulating root growth (Yan *et al.*, 2011). However, despite their known crucial roles in many aspects of plant development and responses to abiotic stress, the functions of the majority of F-box proteins in rice remain to be determined.

In this study, we demonstrated that the expression of *OsFBX322* was downregulated in response to different types of ionizing radiation. Transgenic *Arabidopsis* lines overexpressing *OsFBX322* exhibited increased sensitivity to radiation. We propose that *OsFBX322* is involved in radiation-responsive gene expression in rice and plays a negative role in tolerance to radiation in plants.

## Materials and Methods

### Plant materials and radiation treatment conditions

To examine plant responses to radiation, rice seeds (*Oryza sativa cv*. Ilpoom) were exposed to gamma- irradiation, ion beams, and cosmic rays as described by Hwang *et al.* (2014). Briefly, for gamma-irradiation treatment, mature seeds were exposed to a dose of 200 Gy generated by a ^60^Co gamma irradiator (maximum output, 150 TBq; ACEL, Nordion, Ottawa, ON, Canada) at the Korea Atomic Energy Research Institute. The ion beam treatment consisted of irradiation with 220 MeV carbon ions (LET 107 keV/um), at a dose of 40 Gy, generated by an AVF cyclotron (Japan Atomic Energy Agency, Takasaki, Japan). Exposure to cosmic rays was achieved by placing samples on “Shijian-8”, an unmanned breeding spacecraft, for 15 days. For transcriptional expression analysis, irradiated seeds were cultured on half-strength MS medium and grown for 9 weeks at 24 °C, photoperiod of 16 h light/8 h dark, and 70% humidity.

### Semi-quantitative and quantitative RT-PCR analysis

Total RNA was isolated from transgenic *Arabidopsis* plants and from 3-week-old seedlings germinated from rice seeds using TRIzol reagent, as described by the manufacturer (GibcoBRL, OH, USA). Reverse transcription was performed for 60 min at 42 °C using a Power cDNA Synthesis Kit (Intron Biotech Inc., Sungnam, Korea) with 1 μg of oligo(dT)15 primers and 1 μg of total RNA as template. The resultant cDNA was used as a template for semi-quantitative and quantitative RT-PCR. Semi-quantitative RT-PCR amplification was performed with gene-specific primers and 1 μL cDNA as template (Table 1). The resulting RT-PCR products were analyzed on a 1.0% (w/v) agarose gel with ethidium bromide staining. Quantitative RT-PCR was performed using an Eco Real-Time PCR system (Illumina, CA, USA) with SYBR Premix Ex Taq^TM^ (TaKaRa). The PCR cycle was 95 °C for 10 min, followed by 45 cycles of 95 °C for 10 s and 60 °C for 30 s. Primer sequences are listed in Table 1.

**Table 1:** Primer and probe sequences used for real-time RT-PCR.

cDNA GenBank Accession Nr.	Description	Forward and reverse primers
Os03g0718100	*ACT1*(Actin-1)	5’-TGAAGTGCGACGTGGATATTAG
		5’-CAGTGATCTCCTTGCTCATCC
Os09g0344400	*OsFBX322* (F-Box domain containing protein)	5’-GCTGGTACATGTTCAAACCG
		5’-TGTCGAGGACTAGCAAGGTG
Os09g0324300	*OsFBX313* (F-box domain containing protein)	5’-CGCTTCTTCTCCCTTGACTT
		5’-AGCTCCCATGACCATGAGTT
Os08g0177600	double-strand break repair protein *MRE11*, putative	5’-TCTGCAGAACAGGTTTGGTC
		5’-TTGCAAGCAGAAAGGATGTC
Os07g0633400	Calmodulin-binding protein	5’-TTGGAGAAGGCAAGGAGATT
		5’-ACTTGCCATCCTCAATCACA
Os01g0628000	Cytochrome P450, 72A1	5’-TGTTATCGAGGAGTGCAAGC
		5’-CCCAAACAGACCAAGAACCT

### Multiple sequence alignment and phylogenetic analysis

The rice OsFBX322 sequence was acquired from the National Center for Biotechnology Information (NCBI). Phylogenetic analysis was performed by in MEGA6 software (Tamura *et al.*, 2013) using the default setting. A phylogenetic tree was constructed by the Neighbour-joining (NJ) method (Saitou and Nei, 1987) with 1000 bootstrap replications (Felsenstein, 1985).

### Transient expression of *OsFBX322* in tobacco epidermal leaf cells

*OsFBX322* cDNA was amplified using the PCR primers 5’-ATGGTGAGGAGGAAGTCGAA-3’ and 5’-TTAAGCATCGTCACAAATAC-3’. The *OsFBX322* fragment was cloned into the pCR/GW/TOPO (Invitrogen, CA, USA) entry vector and then sub-cloned into the binary vector pMDC43 for subcellular localization analysis (Curtis and Grossniklaus, 2003). *N. benthamiana* leaves were infiltrated with *A. tumefaciens* GV3101 cells carrying appropriate plasmids. *A. tumefaciens* cells that specifically inhibited plant post-transcriptional gene silencing were co-infiltrated in order to facilitate high expression of recombinant proteins, as previously described by Hernández-Sánchez *et al.* (2015). *A. tumefaciens* cells were grown to ~OD_600_, collected, and re-suspended in a similar volume of infiltration buffer (10 mM MgCl_2_, 10 mM MES (pH 5.6), and 100 μM acetosyringone). An equal mixture of *Agrobacterium* strains containing the translational fusion constructs and p19 plasmid was used for co-infiltration into the abaxial air space of *N. benthamiana* leaves. Expression was visualized 3 days after infiltration. The empty vector was used as a control. The NLS-RFP construct was utilized as a nuclear marker (Lee *et al.,* 2001).

### Generation of transgenic *Arabidopsis*

RNA was extracted from *OsFBX322* (Os09g0344400) seedlings and used to generate cDNA. Primers for RT-PCR amplification incorporated *Xba*I or *Kpn*I restriction sites and were as follows: 5’-tctagaATGGTGAGGAGGAAGTCGA AG-3’ and 5’-ggtaccTTAAGCATCGTCACAAATACAT A-3’. The amplicon was cloned into pGEM-T Easy (Promega, Madison, WI) and subsequently digested with *Xba*I and *Kpn*I (TaKaRa, Tokyo, Japan). The digested fragment was then inserted into pHC21, which was modified from pCAMBIA2300. The recombinant plasmid was introduced into *Agrobacterium tumefaciens* GV3101 and used to transform *Arabidopsis* plants (*Arabidopsis thaliana* ecotypes Landsberg *erecta*) via the floral dip method. Homozygous T_3_ lines were used for further analyses and transgenic plants were maintained as described above.

### Gamma irradiation of transgenic plants at the seed and seedling stage

To determinate radiation sensitivity of seed, seeds of the T_3_ generation from different transgenic lines and WT (*Arabidopsis thaliana* ecotypes Landsberg *erecta*) were exposed to gamma radiation, and then sown on MS medium containing 3% sucrose and 0.25% phyta-gel (pH 5.8) at 22 ^o^C with 16 h light/8 h dark cycle. For radiation sensitivity assay at seedling stage, 14-day-old plants grown in soil were irradiated at 0, 25, 50, and 100 Gy, respectively, for 24 h with a gamma irradiator (ACEL). At day 4 after gamma irradiation, growth rate was determined.

### *In silico* analysis of co-expressed genes

Functional gene interactions were identified in *OsFBX322* using weighted gene co-expression network analysis in R package WGCNA (Langfelder and Horvath, 2008), following the procedure previously described in Hwang *et al.* (2016). The module selected as the *OsFBX322* interacting gene cluster was used to reconstruct a co-expression network in R package ARACNE. The gene-gene interactions detected by ARACNE were visualized using Cytoscape (Shannon *et al.*, 2003).

## Results

### Downregulation of *OsFBX322* expression in response to ionizing radiation

**Figure 1:**
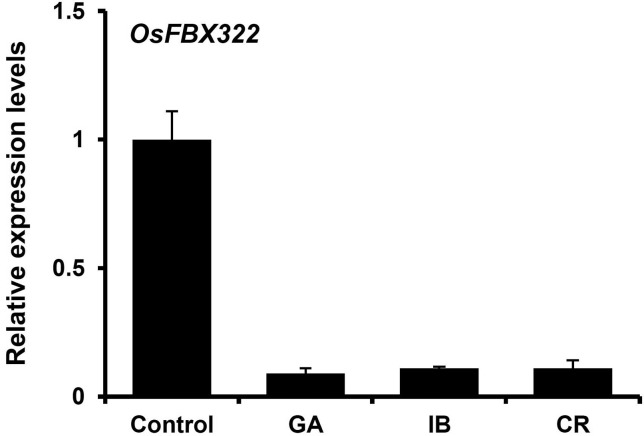
*OsFBX322* expression in 3-week-old rice seedlings after exposure to gamma irradiation, ion beams, or cosmic rays. Quantitative RT-PCR analysis of *OsFBX322* expression after irradiation. The *ACT1* transcript was used for normalization of mRNA content. Transcript levels are displayed relative to *ACT1* mRNA in non-treated plants (control). Control, non-irradiated; GA, 200 Gy gamma-irradiation; IB, 40 Gy carbon ions; CR, exposure on an unmanned spacecraft for 15 days.

The microarray analysis showed that many *FBX* genes including *OsFBX322* were differentially regulated in response to three different types of radiations (Hwang *et al.*, 2014). We confirmed expression level of *OsFBX322* with the treatments of three different radiations (gamma radiation, ion beam, and cosmic ray). Results of quantitative reverse transcription PCR (qRT-PCR) showed that *OsFBX322* was dramatically reduced after exposure to three different types of radiations (Figure 1), indicating that *OsFBX322* shows radiation responsive expression.

### Sequence comparison and phylogenetic analysis of OsFBX322 protein

**Figure 2:**
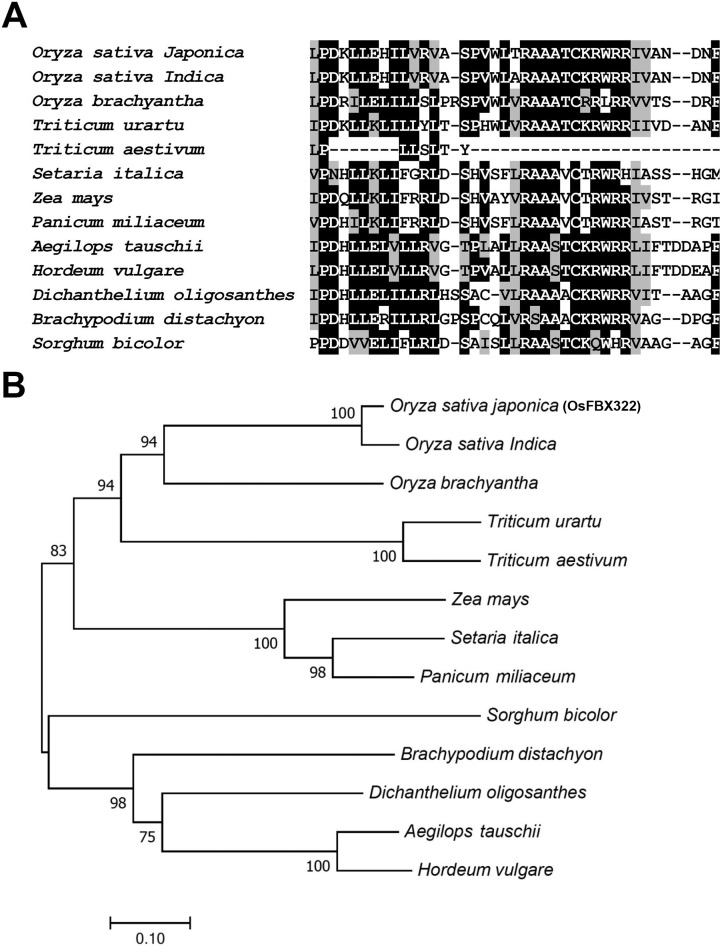
Sequence alignment and phylogenetic tree of OsFBX322 with homologous proteins. (A) Sequence alignment of the conserved F-box domain in OsFBX322 and its homologues. Conserved and similar amino acids are shown in black and grey shading, respectively. (B) Phylogenetic tree analysis OsFBX322 and its homologues. Accession numbers for the respective protein sequences are as follows: *Oryza sativa japonica* (BAT07596.1), *Oryza sativa Indica* (EAZ0874.1), *Oryza brachyantha* (XP_006661142.1), *Triticum urartu* (EMS51354.1), *Triticum aestivum* (CDM87191.1), *Setaria italica* (XP_022680036.1), *Zea mays* (PWZ41408.1), *Panicum miliaceum* (RLN34519.1), *Aegilops tauschii* (XP_020198308.1), *Hordeum vulgare* (BAK05154.1), *Dichanthelium oligosanthes* (OEL21830.1), *Brachypodium distachyon* (KQK06273.1), *Sorghum bicolor* (EER96573.1). The amino acid sequences were aligned and the tree was generated using MEGA 6. The neighbor-joining method with default parameters was used for phylogeny reconstruction. Statistical support for the tree topology was assessed by a bootstrap analysis with 1,000 replications. The values at each node are the bootstrap value.

Sequence analysis using BLASTP revealed that OsFBX322 contains an F-box domain in its N-terminal region, which is a hallmark of F-box proteins from other plant species. The versatile image from the multiple sequence alignment program ClustalX highlighted the conserved features (Figure 2A). The results indicate that the F-box domains were conserved among homologous proteins.

In order to further elucidate the evolutionary relationship between different species, we used the Neighbor-joining algorithm to construct a phylogenetic tree for estimating evolutionary distances and testing evolutionary hypotheses for OsFBX322 based on amino acid residues (Figure 2B). The phylogenetic analysis showed that OsFBX322 is closely related to the *Oryza sativa indica* clade, and this supported that there is a close relationship of *japonica* and *indica* species.

### Nuclear localization of OsFBX322

**Figure 3:**
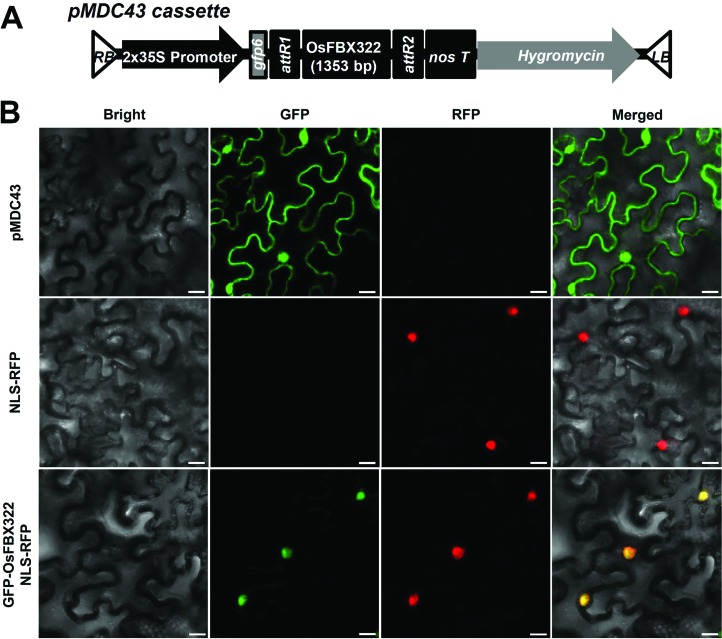
Subcellular localization of OsFBX322 in tobacco epidermal cells*.* (A) Schematic representation of the *GFP-OsFBX322* construct. (B) Detection of GFP fluorescence. *OsFBX322* subcellular localization was investigated using a confocal microscope. Tobacco leaves were transformed with the pMDC43 vector (GFP alone) (upper panels), NLS-RFP (middle panels) or *GFP-OsFBX322* (lower panels). From left to right: bright field, GFP, RFP and merged images. Scale bars: 20 μm.

An N-terminal GFP translational fusion protein was generated to determine the *in vivo* subcellular localization of OsFBX322 (Figure 3A). The *OsFBX322* open reading frame was cloned into the pCR8 entry vector and sub-cloned into the pMDC43 gateway binary vector. Transient expression of GFP-OsFBX322 was examined in *N. benthamiana* leaves infiltrated with *Agrobacterium* carrying the expression plasmid. GFP fluorescence was observed using a laser-scanning confocal microscope. To reveal the subcellular localization of OsFBX322, we used a co-transformation protocol with a GFP-OsFBX322 and NLS-RFP fused construct (NLS-RFP). As shown in Figure 3B, the subcellular distribution of green and red fluorescent signals overlapped, indicating that OsFBX322 was localized to the nucleus.

### Overexpression of *OsFBX322* in transgenic *Arabidopsis*

**Figure 4:**
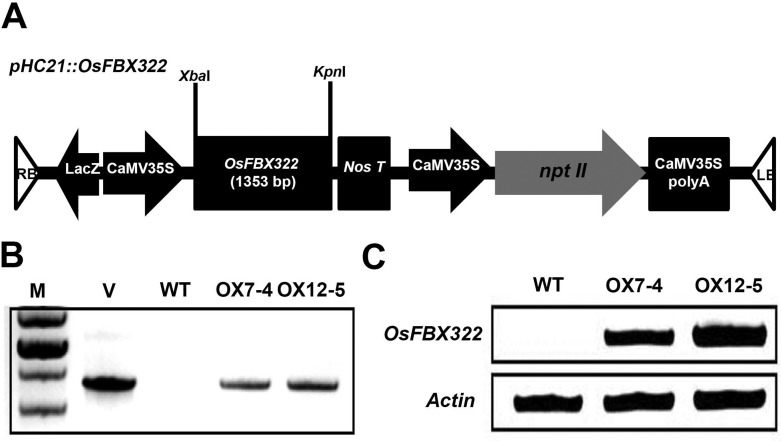
*OsFBX322* overexpression in transgenic *Arabidopsis* plants. (A) Schematic representation of the 35S:*OsFBX322* overexpression construct. *OsFBX322* was regulated by the CaMV35S promoter (CaMV35S). (B) Genomic PCR analysis of untransformed wild-type plants and transgenic *Arabidopsis* lines. The presence of *OsFBX322* was verified by genomic PCR (Table 1). M, molecular mass marker; V, *pHC21::OsFBX322* vector; WT, untransformed wild-type plants; OX7-4 and OX12-5, transgenic 35S:*OsFBX322* lines. (C) RT-PCR analysis of *OsFBX322* transcripts in WT and transgenic plants. *Actin2* was used as a control.

The *in vivo* functions of *OsFBX322* were investigated in *Arabidopsis* transformed with a vector carrying a fusion of the *OsFBX322* cDNA and the CaMV35S promoter (Figure 4A). Fourteen independent T_1_ plants were selected on media containing kanamycin. To determine the *OsFBX322* copy number in transgenic plants, T_1_ plants were self-pollinated and the progeny (T_2_) were allowed to segregate on selection media. Following self-pollination of the T_2_ lines, two T_3_ homozygous lines (OX7-4 and OX12-5) that contained a single T-DNA insertion were selected for analysis (Figure 4B). The presence of *OsFBX322* cDNA (1.353 kb) was verified by genomic DNA PCR using gene-specific primers (Figure 4B). RT-PCR analysis of wild-type plants and the overexpression lines OX7-4 and OX12-5 showed that the integrated *OsFBX322* was strongly detectable only in the OX7-4 and OX12-5 plants (Figure 4C). In order to confirm effect by *OsFBX322* overexpression, we used two independent *OsFBX322*-overexpressing lines (OX7-4 and OX12-5) for further analyses.

### Enhanced sensitivity of transgenic *OsFBX322* overexpressing lines to radiation

**Figure 5:**
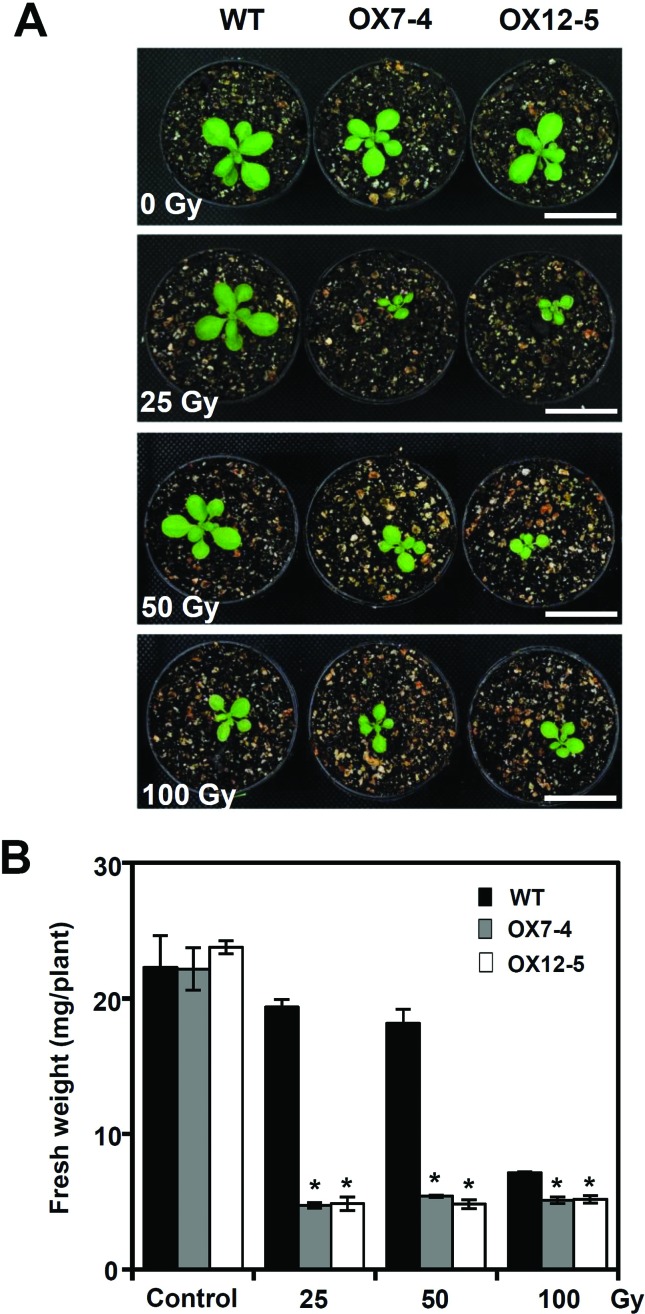
Phenotype analysis of transgenic 35S:*OsFBX322* plants. (A) Enhanced sensitivity of 35S:*OsFBX322* plants. Two-week-old wild-type (WT) and 35S:*OsFBX322* (OX7-4 and OX12-5) plants were exposed to gamma irradiation as indicated. Plants were photographed after 4 days recovery under normal conditions. Scale bar = 2.5 cm. (B) Fresh weights of irradiated WT and transgenic plants. Average fresh weight was estimated from 3–5 plants. Data are means ± SE from at least three independent experiments. Asterisks indicate statistical significance (*p* < 0.05, Student’s *t*-test) of differences between transgenic lines and WT.

The reduction of *OsFBX322* expression after irradiation suggested that *OsFBX322* may play an important role in the radiation response. Therefore, we evaluated the radiation sensitivity of WT and transgenic plants. *OsFBX322*-overexpressing lines, germinated from gamma-irradiated seeds, displayed different rate of germination and early seedling growth after 100 Gy gamma irradiation in comparison with a WT control (data not shown). To determine the direct effect of gamma radiation on the seedling stage, 14-day-old seedlings of transgenic lines were irradiated with 25, 50 and 100 Gy gamma radiation. *OsFBX322* overexpression also improved the radiation sensitivity at the seedling stage (Figure 5). As shown in Figure 5, there were no apparent differences in growth between unirradiated wild-type plants and wild-type plants exposed to 25 or 50 Gy of gamma-irradiation for 24 h. By contrast, growth was inhibited in both the OX7-4 and OX12-5 transgenic lines after the same radiation regime (Figure 5A). The fresh weights of OX7-4 and OX12-5 were also significantly lower than that of the WT (Figure 5B). *OsFBX322*-overexpressing transgenic plants were more sensitive than the WT to radiation. This suggested that overexpression of *OsFBX322* caused increased sensitivity to gamma irradiation.

### Co-expression network analysis to identify *OsFBX322*-related genes

**Figure 6:**
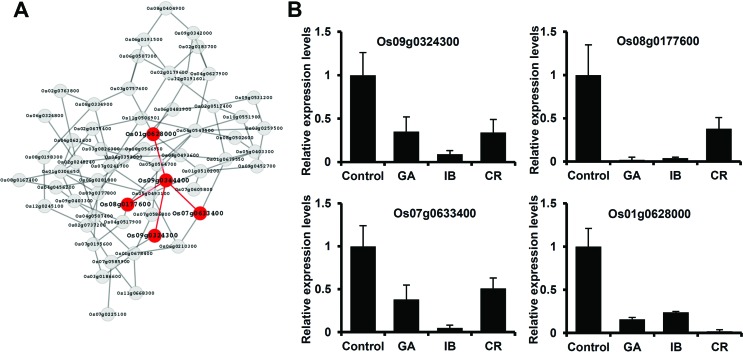
Co-expression relationships between *OsFBX322* and related genes. (A) *OsFBX322* (Os09g0344400) functional gene interactions within a co-expression network constructed by R package ARACNE. Nodes and edges represent the different genes and their functional interactions, respectively. Genes and interactions highlighted in red closely interacted with *OsFBX322* (Os09g0344400). (B) qRT-PCR analysis of co-expressed genes in rice after exposure to GA, IB, or CR. Control, non-irradiated; GA, 200 Gy gamma-irradiation; IB, 40 Gy carbon ions; CR, exposure on an unmanned spacecraft for 15 days.

Co-expression network analysis was used to identify genes linked to *OsFBX322* (Figure 6). Four genes were identified: cytochrome P450 (Os01g0628000), calmodulin-binding protein (Os07g0633400), double-strand break repair protein *MRE11* (Os08g0177600), and F-box domain containing protein *OsFBX313* (Os09g0324300) (Figure 6A). Expression of these genes after radiation exposure was examined using real-time PCR (Figure 6B). Compared to the unirradiated control, all four genes exhibited lower expression levels in response to gamma-irradiation, ion beams, and cosmic rays. Although expression of individual genes varied, all four genes exhibited a downregulated response (Figure 6B). These results suggested that the co-expressed genes with *OsFBX322* had similar functions in the response to multiple types of irradiation.

## Discussion

The mechanisms underlying differential responses to radiation are likely based on differences at the DNA level. This could include increased mutation rates in essential genes, which may lead to higher or lower sensitivities to radiation. Our previous DNA microarray analysis identified 53 candidate genes that were up- or downregulated in response to radiation exposure, but that were not differentially regulated by other stresses (Hwang *et al.*, 2014). One of these genes, *OsFBX322*, was substantially downregulated after exposure to gamma-irradiation, ion beams, or cosmic rays (Hwang *et al.*, 2014). Attempts have also been made to address the biological functions of the *OsFBX322*, we have generated overexpression transgenic plants in *Arabidopsis*. Our analysis of this transgenic plant has new and important information response to radiation condition.

F-box domain containing proteins belong to a versatile group and have multiple functions in degradation of cellular proteins, developmental processes, and responses to abiotic stress (Lechner *et al.*, 2006; Dreher and Callis, 2007; Zhang *et al.*, 2008). However, the molecular mechanisms by which these proteins exert their functions are not fully known. In our semi-quantitative and quantitative results, the expression levels of *OsFBX322* were found to be downregulated after gamma-irradiation, ion beam and cosmic-ray exposure (Figure 1). This is consistent with previous microarray results (Kim *et al.*, 2013; Hwang *et al.*, 2014) and indicates that *OsFBX322* may play a functional role in the response to radiation. Additionally, we examined the subcellular localization of OsFBX322 using a GFP translational fusion. Transiently-expressed OsFBX322 was found in *N. benthamiana* epidermal cells (Figure 3). Previous reports showed that F-box proteins were localized in various intracellular compartments including the nucleus, cytosol, vacuole, and chloroplasts (Kuroda *et al.*, 2012). In rice, MOF (a putative F-box protein) interacted with SKP1-like protein OSK1, suggesting that MOF was a component of SCF ubiquitin and regulated cellular functions via ubiquitin-mediated protein degradation or via signaling pathways in the nucleus (He *et al.*, 2016). OsFBX322 exhibited nuclear localization and may play a similar role to that of MOF. *OsFBX322* was overexpressed in *Arabidopsis* to further investigate its radiation sensitivity (Figure 4). *OsFBX322* overexpressing transgenic plants showed increased sensitivity to gamma irradiation compared to control plants (Figure 5). These results indicate that *OsFBX322* is involved in the plant response to radiation and that *OsFBX322* is a radiation responsive gene.

Highly correlated gene expression profiles are suggestive of common regulatory mechanisms and similar biological functions. Detection of gene modules in a co-expression network can facilitate the discovery of biologically meaningful clusters, and these methods were successfully used in various biological contexts (Langfelder and Horvath, 2008; Hwang *et al.*, 2016). Recent studies demonstrated that co-expression networks could be used to identify a set of candidate genes underlying specific phenotypes (Mutwil *et al.*, 2010, Ficklin and Feltus, 2011). In this study, module detection was used to identify biological systems in rice that changed significantly in response to ionizing radiation (Figure 6). Four genes co-expressed with *OsFBX322* were identified: Os08g0177600 (double-strand break repair protein MRE11), Os07g0633400 (Calmodulin-binding protein), Os01g0628000 (Cytochrome P450, 72A1), and an F-box protein, Os09g0324300 (*OsFBX313*). *OsFBX322* was also classified as an F-box protein. Recent research indicated that F-box domain proteins had important roles in regulating various developmental processes and stress responses (Lechner *et al.*, 2006; Dreher and Callis, 2007; Zhang *et al.*, 2008). Expression of F-box proteins in rice led to a reduced tolerance of abiotic stresses (Yan *et al.*, 2011) and F-box gene expression was downregulated by ionizing radiation (Hwang *et al.*, 2016). This study provides evidence that a number of F-box protein-encoding genes are likely to be involved in radiation responses. Additionally, previous studies showed that MRE11, as part of a complex with the Rad50 and Xrs2/Nbs1 proteins, functioned in diverse DNA repair and metabolic mechanisms after induction by ionizing radiation in rice (Hong *et al.*, 2005; Ji *et al.*, 2012). The gene encoding putative OsMRE11-like protein (Os08g01776600) was co-expressed with *OsFBX322* and was downregulated in response to ionizing radiation (Figure 6). This suggested that OsMRE11-like protein might perform essential DNA repair and metabolic functions after ionizing radiation exposure in rice. Calmodulin-binding protein and cytochrome P450 were shown to be involved in the stress responses (Yang and Poovaiah, 2002; Narusaka *et al.*, 2004). The presence of multiple modules with connectivity suggests that the several regulatory networks may be present in controlling unique and/or overlapping sets of *OsFBX322* genes. The high level of connectivity with FBX family gene and several stress response genes led us to hypothesize a strong likelihood of combinatorial and synergistic regulation to radiation response by these genes. However, further research is required to confirm these genes identified by transcriptional analysis.

Our study demonstrates that *OsFBX322* plays a significant role in the radiation responsive pathway, and corresponding genes in other species may be a valuable resource for producing radiation-sensitive transgenic crop plants. Such transgenic plants may also be useful for the detection of radiation sensitivity.
